# A recurrent mutation in *GUCY2D* associated with autosomal dominant cone dystrophy in a Chinese family

**Published:** 2011-12-15

**Authors:** Xueshan Xiao, Xiangming Guo, Xiaoyun Jia, Shiqiang Li, Panfeng Wang, Qingjiong Zhang

**Affiliations:** State Key Laboratory of Ophthalmology, Zhongshan Ophthalmic Center, Sun Yat-sen University, Guangzhou, China

## Abstract

**Purpose:**

To identify the genetic locus and mutation responsible for autosomal dominant cone dystrophy (adCOD) in a large Chinese family and to describe the phenotypes of the patients.

**Methods:**

Genomic DNA and clinical data were collected from the family. Genome-wide linkage analysis was performed to map the disease locus, and Sanger dideoxy sequencing was used to detect the mutation in a candidate gene.

**Results:**

Initially, genome-wide linkage analysis mapped the disease to 17p13.1 between D17S831 and D17S799, with a maximum lod score of 2.71 for D17S938 and D17S1852 at theta=0. Sequence analysis of the guanylate cyclase 2D gene (*GUCY2D*) in the linkage interval detected a recurrent heterozygous mutation, c.2513G>A (p.Arg838His). This mutation was present in all eight patients with adCOD, but neither in any of the six unaffected family members nor in 192 control chromosomes.

**Conclusions:**

adCOD in this family is caused by a recurrent mutation in *GUCY2D*. adCOD can be detected in the first few years after birth in the family by fundus observation and electroretinogram recordings.

## Introduction

Cone dystrophy (COD) is a retinal disease characterized by the dysfunction or degeneration of cone photoreceptors that are responsible for central and color vision. COD can be classified into two major types: stationary and progressive. Progressive COD might be difficult to differentiate from cone-rod dystrophy (CORD), since some degree of rod dysfunction develops in the advanced stage of COD [1,2]. It is suggested that COD represents retinal diseases with predominant cone dysfunction with late onset and mild rod involvement, while CORD indicates retinal degeneration with early onset cone dysfunction followed shortly thereafter by significant rod dystrophy [1]. Patients with progressive COD may complain of photophobia, decreased visual acuity, and color vision defects. Fundus changes may be nearly normal or subtle in the early stage. In the advanced stage, macular degeneration can be observed under an ophthalmoscope.

COD can be inherited as an autosomal dominant (adCOD) [3], autosomal recessive (arCOD) [4], or X-linked trait (xlCOD) [5], although it occurs sporadically in most cases. At least five loci have been designed for COD, namely COD1 (OMIM 304020) [5,6], COD2 (OMIM 300085) [7], COD3 (OMIM 602093) [3], COD4 (OMIM 613093) [4], and COD5 (OMIM 303700) [8]. Mutations in several genes have been identified to be responsible for COD, including guanylate cyclase activator 1A (*GUCA1A*) [3], alpha-prime cone cGMP-specific phosphodiesterase subunit (*PDE6C*) [4], retinitis pigmentosa GTPase regulator (*RPGR*) [6], red and green visual pigment genes (*OPN1LW* and *OPN1MW*) [8], and guanylate cyclase 2D (*GUCY2D*) [9,10]. However, no mutation in *GUCY2D* has been reported in Chinese patients with COD or CORD.

In the present study, progressive COD was found in a three-generation Chinese family with eight affected individuals. A genome-wide linkage study mapped the COD locus to 17p13.1. Sequencing the candidate gene in the linkage interval identified a recurrent c.2513G>A (p.Arg838His) mutation in *GUCY2D* (OMIM 600179).

## Methods

### Family with cone dystrophy

adCOD was identified in a three-generation family living in a small town in Guandong province, China. Eight patients and six unaffected individuals in the family participated in this study. Written informed consent was obtained from the participating individuals or their guardians before the collection of clinical data and genomic samples. This study was approved by the Internal Review Board of the Zhongshan Ophthalmic Center, Sun Yat-sen University, Guangzhou, China and followed the tenets of the Declaration of Helsinki and the Guidance of Sample Collection of Human Genetic Diseases (863-Plan) of the Ministry of Public Health of China. Genomic DNA was prepared from venous leukocytes.

### Genotyping and linkage analysis

Genotyping for 14 family members was performed using 5′-fluorescently labeled microsatellite markers, as previous described [11]. Briefly, a genome-wide scan was performed using panels 1 to 27 of the ABI PRISM linkage Mapping Set Version 2 (Applied Biosystems, Foster City, CA). PCR was conducted at 94 °C for 8 min, followed by 10 cycles of amplification at 94 °C 15 s, 55 °C 15 s, and 72 °C 30 s; then 20 cycles at 89 °C 15 s, 55 °C 15 s, 72 °C 30 s; and finally at 72 °C for 10 min. After mixing with GENESCAN^TM^ 400HD [ROX^TM^] standard (Applied Biosystems) and deionized formamide, the amplicons were denatured at 95 °C for 5 min and then immediately placed on ice for 5 min. The amplicons were separated on an ABI 3100 Genetic Analyzer (Applied Biosystems). Genotyping data were analyzed using the Gene Mapper version 3.5 software package (Applied Biosystems). Two-point linkage analysis was performed by using the MLINK program of the FASTLINK implementation of the LINKAGE program package [12,13]. COD in the family was analyzed as an autosomal dominant trait with full penetrance and with a disease-gene allele frequency of 0.0001. Haplotypes were generated using the Cyrillic 2.1 program (Cyrillic Software, Wallingford, UK) and confirmed by inspection.

### Mutation identification in *GUCY2D*

PCR was used to amplify the genomic fragments of *GUCY2D*. Primers employed to amplify the 19 coding exons and their adjacent intronic region of *GUCY2D* were the same as those previously reported [14]. PCR amplifications were performed in 20 µl reactions containing 80 ng genomic DNA. Touchdown PCR amplification consisted of a denaturizing step at 95 °C for 5 min, followed by 35 cycles of amplification (at 95 °C for 30 s; at 64–57 °C for 30 s, starting from 64 °C and decreasing by 0.5 °C with every cycle for 14 cycles until remaining at 57 °C for 21 cycles; and at 72 °C for 40 s) and a final extension at 72 °C for 10 min. The nucleotide sequences of the amplicons were determined with the ABI BigDye Terminator cycle sequencing kit v3.1 (Applied Biosystems), electrophoresed on an ABI3100 Genetic Analyzer (Applied Biosystems), and analyzed with Seqman software (Lasergene 8.0; DNASTAR, Madison, WI). Any variant detected was initially confirmed by bidirectional sequencing and then evaluated in 192 control chromosomes of 96 normal individuals. Mutation description followed the recommendation of the Human Genomic Variation Society (HGVS).

## Results

**Figure 1:**
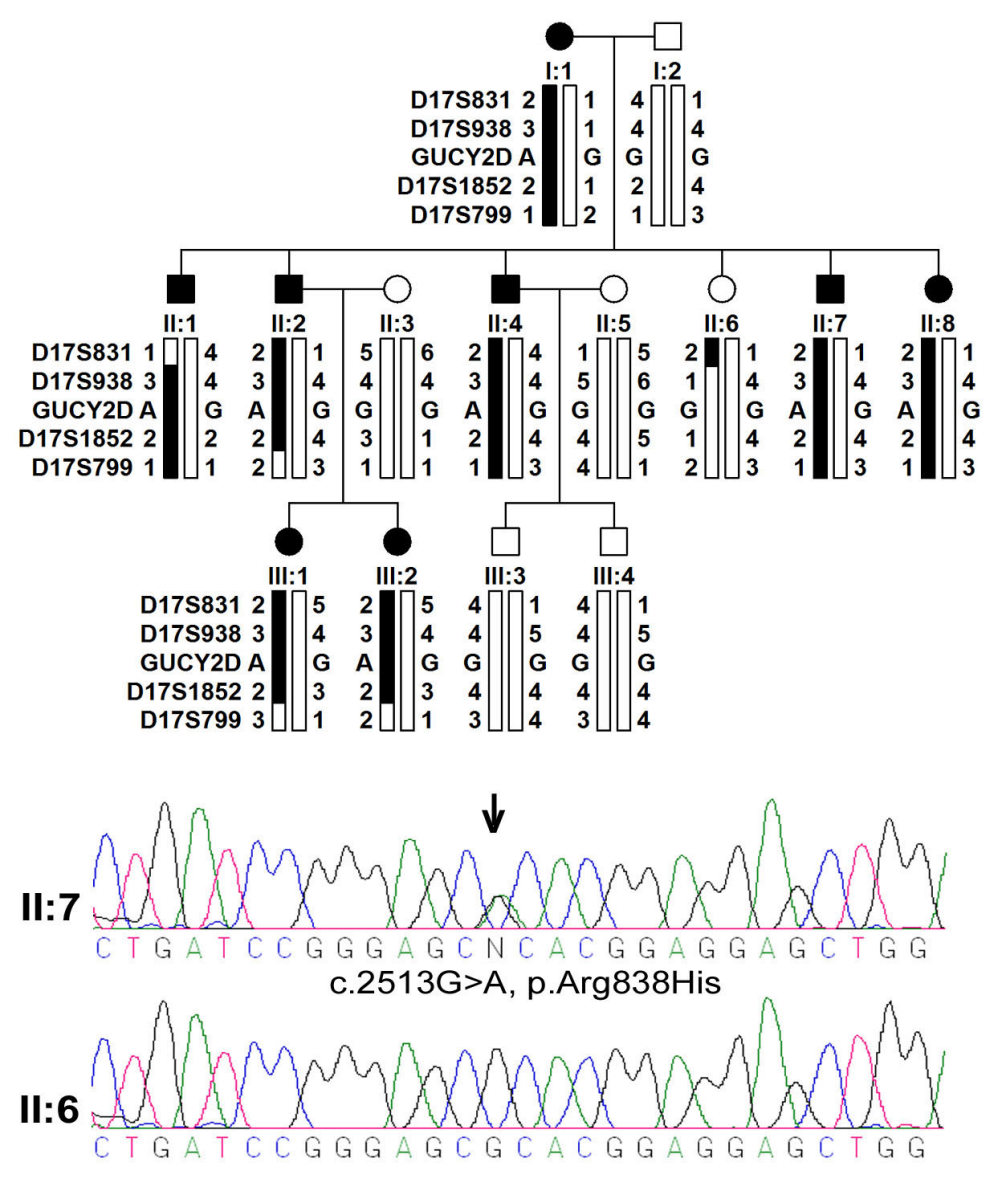
Pedigree, genotyping haplotypes on 17p13.1, and *GUCY2D* mutation. Pedigree and haplotypes are shown at the top. Filled squares (male) or circles (female) represent individuals affected with cone dystrophy. Bars filled with black indicate a disease-associated allele. The sequence chromatograph of *GUCY2D* is shown at bottom, in which the double peaks (arrow indicated) demonstrated the heterozygous recurrent mutation in *GUCY2D*.

**Figure 2:**
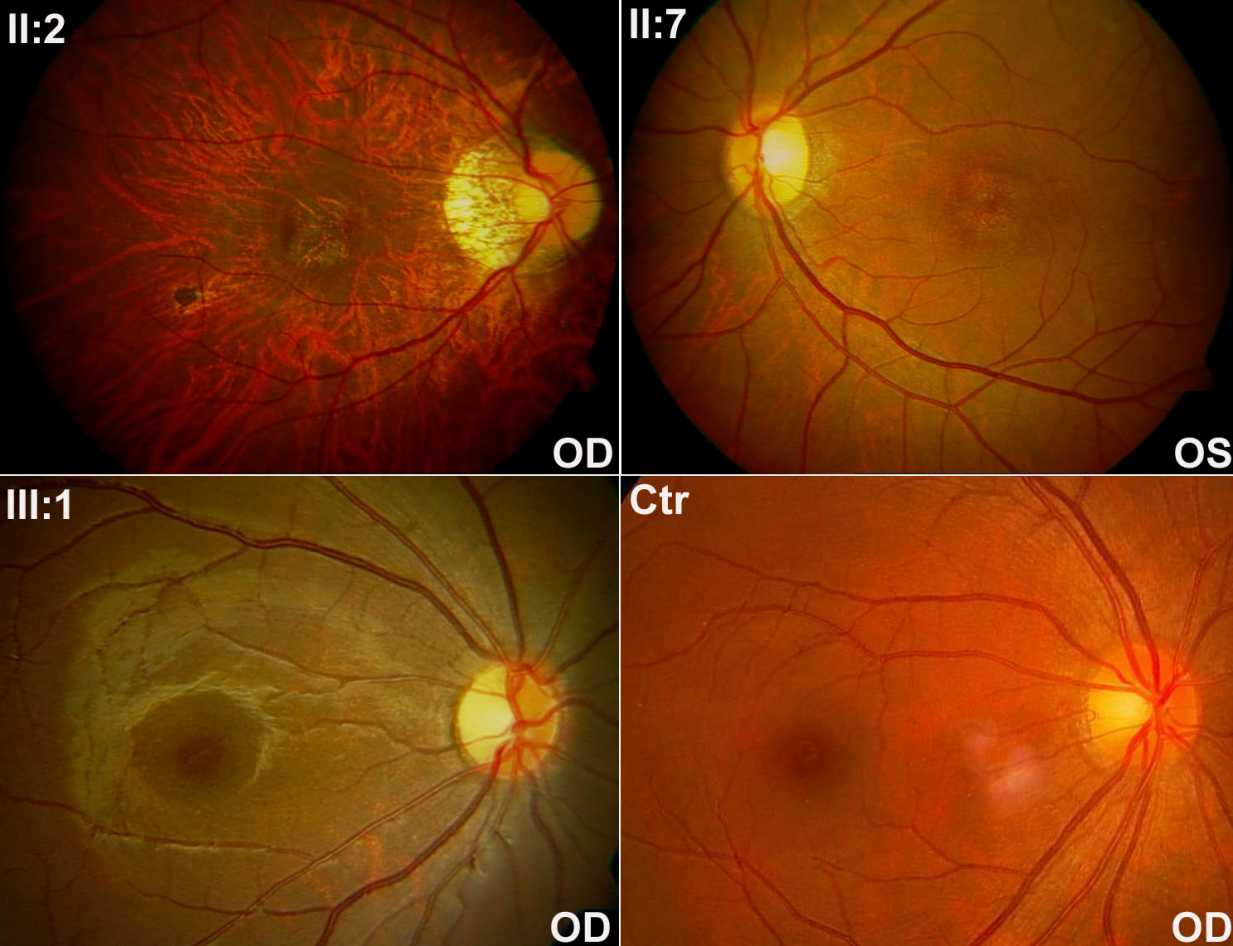
Fundus photos of three patients and a normal control. II:2 and II:7 demonstrated the typical macular atrophy observed in the six adult patients. III:1 at two and half years old showed mild granular retinal pigment epithelial changes, with the tiny yellowish-white deposits in the macula observed in the two youngest patients. Temporal pallor of the optic disc was observed in patients II:2, II:7, and III:1. Mild artery attenuation was noticed in II:2 and II:7. Ctr: fundus photo of a normal control.

**Figure 3:**
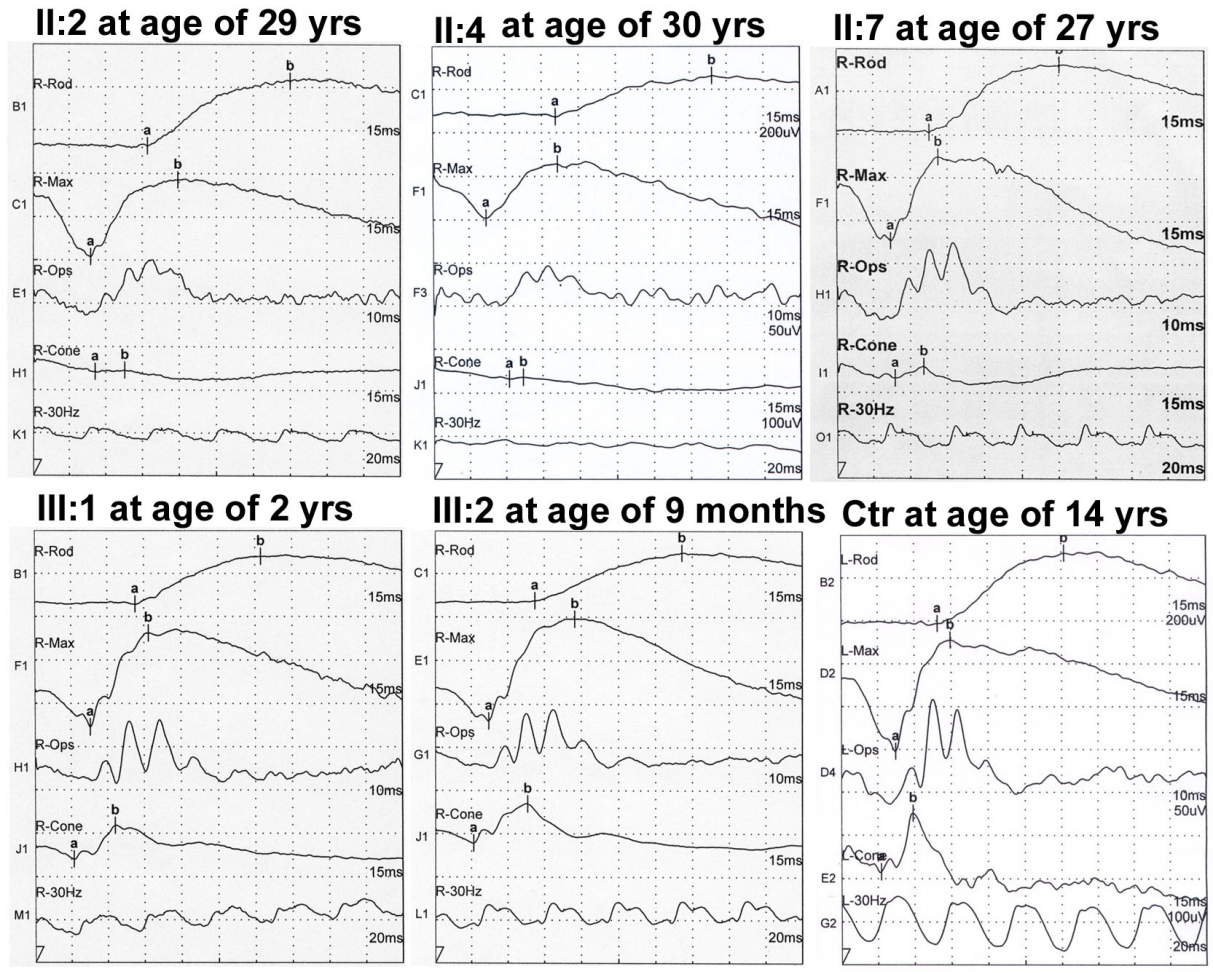
Electroretinography of five patients and a normal control. Reduced photopic responses, abnormal 30 Hz ERG, and normal scotopic Electroretinography (ERG) were shown in all five patients with ERG recordings (II:2, II:4, II:7, III:1, and III:2). Ctr: A normal control.

Fourteen individuals in the family participated in this study (Figure 1). The disease in the family had been passed in three generations. Eight individuals in the family were considered to be affected with adCOD based on clinical information (Table 1; Figure 2, and Figure 3).

**Table 1:** Clinical data of the patients in the Chinese family with an Arg838His mutation in GUCY2D.

**ID#**	**Gender**	**Age (years) at**		**First symptom**	**Visual acuity**	**Refraction (diopters)**	**Fundus changes**	**ERG responses**		**Color vision**
		**exam**	**onset**					**Cones**	**Rods**	
I:1	F	54	Early childhood	photophobia	CF; CF*	−10.0; −10.0	macular atrophy	N/A	N/A	N/A
II:1	M	31	Early childhood	photophobia	0.04; 0.04	−11.0; −10.0	macular atrophy	N/A	N/A	abnormal
II:2	M	29	8 years	photophobia	0.05; 0.1	−8.75; −7.00	macular atrophy	reduced	normal	abnormal
II:4	M	28	7 years	photophobia	0.03; 0.03	−7.00; −4.75	macular atrophy	reduced	normal	abnormal
II:7	M	26	Early childhood	photophobia	0.05; 0.04	−2.75; −0.25	macular atrophy	reduced	normal	abnormal
II:8	F	19	6yrs	photophobia	0.1; 0.1	−6.00; −7.00	macular atrophy	N/A	N/A	abnormal
III:1	F	2.5	N/A	no symptom	N/A	+0.25; plano	macular white spots	reduced	normal	N/A
III:2	F	0.75	N/A	no symptom	N/A	−2.25; −1.00	macular white spots	reduced	normal	N/A

Note: *This patient had horizontal nystagmus and mild cortical opacity of both lenses.

Two young affected girls (III:1 and III:2) in the family, aged at two and a half years and nine months, respectively, had no visual symptom at the time of examination (Figure 1 and Table 1). However, fundus observation revealed carpet-like changes with multiple fine yellowish-white spots in the macular region, as well as pallor in the temporal optic discs of these two girls (Figure 2). Electroretinography (ERG) recordings demonstrated reduced cone responses that confirmed the clinical findings (Figure 3).

For the other six adult patients, all had photophobia and blurred vision noticed at about 6 to 8 years old, with progressively decreased visual acuity thereafter. Night vision in these patients was well preserved. Examination of Ishihara color plates on 5 patients suggested color vision defects. Macular atrophy and temporal pallor of the optic disc were present in all six adult patients (Figure 2). Cone responses were significantly reduced in three adult patients who received ERG examination (Figure 3). Rod responses were normal in all five patients (three adults and two young girls), as measured by ERG recordings (Figure 3).

The genome-wide linkage scan excluded linkage to other regions and mapped the adCOD to 17p13.1 between D17S831 and D17S799, with a maximum lod score of 2.71 at theta=0 (Figure 1, Table 2). One gene known to cause CORD, *GUCY2D*, was present in the linkage interval. Subsequently, sequencing analysis of *GUCY2D* detected a recurrent heterozygous mutation, c.2513G>A (p.Arg838His). This mutation cosegregated with adCOD in the family with a maximum lod score of 2.71, but was not present in six unaffected family members or in 192 control chromosomes.

**Table 2:** Two-point lod scores of family for markers around *GUCY2D*.

	**Position**		**Lod score at theta=**						
**Markers**	cM*	**Mb#**	**0**	**0.01**	**0.05**	**0.1**	**0.2**	**0.3**	**0.4**
D17S849	0.6	0.43	-inf	−3.61	−1.61	−0.82	−0.17	0.07	0.11
D17S831	6.6	1.91	-inf	−1.33	−0.07	0.35	0.56	0.50	0.30
D17S938	14.8	6.25	2.71	2.67	2.49	2.25	1.74	1.16	0.53
*GUCY2D*		7.91	2.71	2.67	2.49	2.25	1.74	1.16	0.53
D17S1852	23.2	10.52	2.71	2.67	2.49	2.25	1.74	1.16	0.53
D17S799	32.8	13.17	-inf	−1.33	−0.07	0.34	0.54	0.44	0.22
D17S921	37.3	14.26	-inf	−1.62	−0.35	0.09	0.33	0.29	0.14

Genethon, # Homo genome (Build 37.2) Chr1 Primary_Assembly

## Discussion

We identified a Chinese family with eight patients showing signs of adCOD that was transmitted as autosomal dominant trait in the family. A genome-wide linkage analysis mapped the disease to 17p13.1 between D17S831 and D17S799. Subsequent mutational screening of a candidate gene in the linkage interval identified a recurrent heterozygous c.2513G>A (p.Arg838His) mutation in *GUCY2D*. This mutation was present in all eight patients in the family, but was absent in unaffected family members and normal controls. All of these lines of evidence support the view that a mutation in *GUCY2D* is responsible for the adCOD in the family.

To date, at least 125 mutations in *GUCY2D* have been identified to be responsible for retinal degeneration, including Leber congenital amaurosis, CORD, COD, and retinitis pigmentosa, based on HGMD® Professional 2011 accessed as of June 24, 2011. A review of the original reports revealed that 10 of the 125 mutations were associated with COD or CORD in 33 families (Table 3). For the 10 mutations in the 33 families, codon 838 accounted for six mutations (66.7%) in 29 families (90.6%). Up to the present, all patients with *GUCY2D* mutations at codon 838 exhibited COD or CORD except for one patient, who had c.2513G>C (p.Arg838Pro) and Leber congenital amaurosis, but no clinical details were present [15]. It is unclear if there is any functional difference among these different mutations involving codon 838.

**Table 3:** Reported *GUCY2D* mutations associated with cone or cone-rod dystrophy.

**No.**	**Nucleotide change**	**Residue change**	**Families**	**Phenotypes**	**References**
1	Unclear	P575L	1	adCOD*	[17]
2	2511_2512delGCinsCA	[Glu837Asp,Arg838Ser]	1	adCORD	[10,21]
3	2511_2516delGCGCACinsCTGCAT	[Glu837Asp,Arg838Cys,Thr839Met]	1	adCORD	[22]
4	2512C>T	Arg838Cys	16	11/adCORD	[9,10,18,21,23–25]
				5/adCOD	[9]
5	2512C>G	Arg838Gly	1	adCORD	[9]
6	2513G>A	Arg838His	9	5/adCORD	[21,24–27]
				4/adCOD	[9,16]
7	2513G>C	Arg838Pro	1	1/adCORD	[27]
8	2846T>C	Ile949Thr	1	arCORD	[28]
9	2540_2542delAGAinsTCC	[Gln847Leu,Lys848Gln]	1	adCORD	[29]
10	2744_2749delTCATTGinsCCATTC	[I915T,G917R]	1	adCORD	[24]

Of the 33 families with *GUCY2D* mutations, 22 had adCORD, 1 had arCORD, and 10 had adCOD. For the 10 adCOD families, 5 had the c.2512C>T (p.Arg838Cys) mutation [9], 4 had the c.2513G>A (p.Arg838His) mutation [9,16], and 1 had p.P575L mutation [17]. The phenotypes of the patients were essentially similar but the ages at onset varied significantly. In the Chinese family, photophobia and poor vision was the initial symptom presented at age around 6 to 8 years old, which appeared earlier than those patients with the p.Arg838His mutations in previously reported families. In addition, the visual acuity for the Chinese patients was generally worse than that for patients in previous reports. The rod function in the Chinese patients was well preserved. Our study provided clinical phenotypes for the two youngest patients at ages less than two and half years old, while previous studies only provided phenotypes for patients over 10 years old.

Except for the common signs and symptoms for COD or CORD, myopia has been reported as a common sign in several families, such as myopia greater than 5D in at least one eye in 18 of 29 patients (62%) in a family with the p.Arg838Cys mutation [18], myopia greater than 6D in all 10 patients of two families with the p.Arg838His or Arg838Cys mutation [19], and moderate myopia in patients of a family with the p.[Glu837Asp, Arg838Ser] mutation [20]. In this study, high myopia was present in five of the eight patients.

In summary, adCOD was identified in a Chinese family and was caused by a recurrent mutation in *GUCY2D*. This is the first report of *GUCY2D* mutation–associated adCOD in the Chinese population. Fundus and ERG changes in the two patients less than three years old provide the earliest signs for adCOD, which would be valuable in clinical diagnosis.
